# Unlocking oncolytic virotherapy potential: leveraging bacteriophages to conquer immunosuppression

**DOI:** 10.3389/fmicb.2026.1957551

**Published:** 2026-10-21

**Authors:** Bilel Boulfrad, Anis Larbi

**Affiliations:** 1 American School of Doha, Doha, Qatar; 2 Department of Medicine, Faculty of Medicine and Health Sciences, University of Sherbrooke, Sherbrooke, QC, Canada; 3 Beckman Coulter Life Sciences, Indianapolis, IN, United States

**Keywords:** bacteriophage, cancer, coating technologies, immunosuppression, oncolytic virotherapy

## Abstract

Oncolytic virotherapy (OVT) offers great potential as an immunotherapy by lysing cancer cells and inducing immunogenic cell death, inciting an anti-tumor immune response. However, immunosuppressive tumor microenvironments (TMEs) in “cold” solid tumors limit OVT effectiveness by reducing viral infiltration and immune responses. Bacteriophages—safe, manipulable bacterial viruses—and their derived mechanisms are emerging as promising tools to address this gap by minimizing TME immunosuppression, maximizing anti-tumor immune activation, and improving overall precision and effectiveness. Bacteriophage characteristics such as their repetitive peptide presentation, biological barrier degradation, and superior payload delivery capabilities can be harnessed for OVT to improve therapy effectiveness. This review explores how engineered OVT can leverage such bacteriophage-derived components through two main approaches. The established synergistic approach allows chimeric vectors such as AAVP to integrate phage safety, cargo capabilities, and precision into OVT. The proposed modular approach aims to transfer specific desirable traits from phages to replication-capable OVs. For example, through novel AI and genetic engineering technology, the first mammalian encoded hyaluronate lyase can be synthetically created. The products of this traditionally prokaryotic enzyme avoid the tumor-progressing effects of current mammalian extracellular matrix-degrading hyaluronate lyases.

## 1 Introduction

Cancer remains a leading global cause of mortality, with the World Health Organization reporting 10 million deaths in 2022, accounting for 1 in 6 deaths that year (WHO Cancer Factsheet, 2026). Cancer incidence and mortality are not slowing down. In fact, cancer cases are projected to reach 35 million in 2050 (a 77% increase compared to 2022). In addition, mortality rates are set to increase by 90%, reaching 18.5 million deaths in 2050 (Bizuayehu et al., 2024). This creates a rising demand for innovative immunotherapies amid rising resistance and heterogeneity. Of the different immunotherapies that harness the body’s immune system to fend off cancer, oncolytic virotherapy is set apart by its combination of direct lysis and immunogenic cell death (ICD), stimulating a localized long-term immune response while relatively minimizing side effects (Galluzzi et al., 2017; Chen et al., 2023). OVT is also characterized by its potential use in combination therapy—along with other immunotherapies or cancer treatments such as radiation or chemotherapy for enhanced combined effectiveness as well as a potential for being engineered for specific cancer types and personalized treatments (Jhawar et al., 2023; Wang, Y. et al., 2024). However, despite its promise, OVT does have its shortcomings. OVT faces both systemic barriers, including immune clearance and off-target infection (Alwithenani et al., 2025), and tumor microenvironment (TME)-specific challenges, including delivery barriers, heterogeneity, and immunosuppression (Volovat et al., 2024), all of which reduce the subset of patients who would positively respond to immunotherapies. In this review, we will explore the potential for bacteriophage-derived mechanisms to counter these challenges, enhancing OVT performance in solid tumors.

## 2 Oncolytic virotherapy: immune reprogramming

Oncolytic virotherapy is an emerging therapeutic approach that leverages genetically engineered or naturally occurring viruses to selectively replicate in and destroy tumor cells while leaving healthy tissues unharmed (Ahsan et al., 2025; Kim and Cho, 2022). This immunotherapy is differentiated as a “living drug,” using a pathogen’s replicative machinery to dynamically fight the tumor from within. Moreover, OVT is distinguished by its unique dual-action mechanism: direct oncolysis during viral replication, followed by a targeted activation of anti-tumor immunity that outlasts viral presence (Alwithenani et al., 2025). This combination of immediate targeted cytotoxicity with long-term adaptive immune responses allows OVT to be an effective immunotherapy (Figure 1).

**Figure 1:**
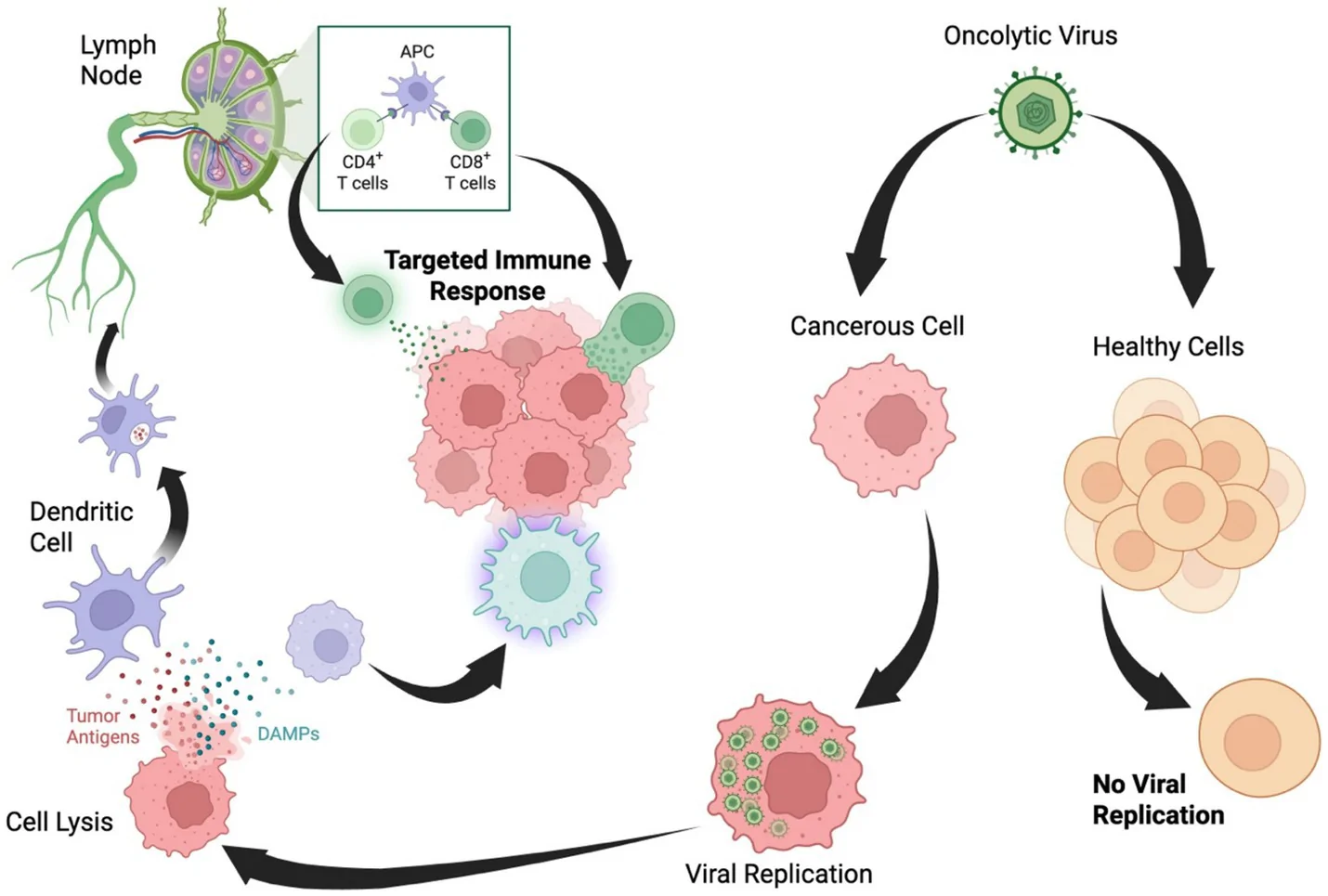
Schematic illustration of the selective replication and immunogenic lytic capabilities of oncolytic viruses. Created in BioRender. Boulfrad (2026). https://BioRender.com/660g2vm.

### 2.1 Mechanisms of action

The capability of oncolytic viruses (OVs) to specifically target tumor cells while sparing healthy ones is a keystone of OVT. Early generations of OVs relied on natural tumor tropism. They are naturally disposed to have a high affinity for tumor-specific receptors that are overexpressed in tumor cells due to dysregulation of certain pathways. An example of this is poliovirus’s natural receptor, CD155, which is overexpressed on the surfaces of a wide range of cancerous cells and promotes the invasion and metastasis of tumors (Gao et al., 2017). This allows poliovirus to selectively target and kill such cancer cells. Moreover, advances in molecular virology and genetic engineering have enabled a diverse range of OVs to target receptors that are prevalent in specific cancer types. An elegant demonstration of this is seen in the viral retargeting of herpes simplex virus 1 (HSV-1) when modified with the P-V528LH adapter, a bi-soluble “bridge” that allows for HSV-1 to bind to the tumor-specific epidermal growth factor receptor (EGFR) rather than its natural nectin-1 target (Lin et al., 2023). This provides the opportunity for the use of HSV-1—which can uniquely accommodate large transgene insertions—in tumors where EGFR is overexpressed, such as breast and colorectal cancer. However, many such “tumor-specific” receptors are present in certain healthy cells too, albeit in lower amounts. To account for this, OV selectivity has also been integrated into viral replication. Many cancers lack specific antiviral defenses or exhibit a dysregulation of signaling pathways. Moreover, specific tumor-selective promoters, such as survivin, can promote unchecked cell-cycle progression and prevent apoptosis. OVs can be engineered to exploit such vulnerabilities with promoter-driven or pathway-dependent replication strategies (Xiao et al., 2026).

### 2.2 Induction of immunogenic cell death

The key to the ability of OVs to induce an anti-cancer immune response lies in how they kill cancerous cells. As illustrated in Figure 1, target cells are lysed to release their contents. This is a form of immunogenic cell death (ICD), which plays a crucial role in the effectiveness of OVT as a treatment. This is because, along with new OVs, damage-associated molecular patterns (DAMPs) are released into the extracellular space. DAMPs include factors such as high mobility group box 1 (HMGB1), mitochondrial DNA (mtDNA), calreticulin, and ATP (Wu et al., 2024; Wang et al., 2025). The release of such signals expresses the need for an immunological response and activates the release of chemokines and pro-inflammatory cytokines that guide and facilitate the mobilization of immune cells to the tumor site. Moreover, ICD also triggers a much longer-lasting and more specialized adaptive immune response through the release of Tumor Associated Antigens (TAAs). Once antigen-presenting cells (APCs) such as dendritic cells reach the tumor, they endocytose TAAs, which can then be used to initiate a specific response against the tumor. The APCs then migrate to lymph nodes where they present the TAAs to cytotoxic (CD8+) and helper (CD4+) T-cells in addition to B-cells through their major histocompatibility complex (MHC) (Lee et al., 2025; Wang et al., 2025). After activation and proliferation, the tumor-specific lymphocytes travel through the bloodstream to infiltrate the tumor, where they mount a targeted attack.

### 2.3 Approved oncolytic viruses

The clinical promise of OVT has been demonstrated through trials of the only FDA-approved replicating OV to date, Talimogene laherparepvec. This melanoma-targeting OV is a modified HSV-1 with a deleted virulence gene to improve safety. It is also armed with the gene for a human cytokine, granulocyte-macrophage colony-stimulating factor (GM-CSF), which is a powerful recruiter of dendritic cells and attracts them to the tumor site. Its use as a neoadjuvant monotherapy in difficult-to-resect cutaneous basal cell carcinomas yielded a 55.6% objective response rate with 50% of patients reaching surgical resection without requiring reconstructive surgery (e.g., skin flaps or grafts; Ressler et al., 2025). Moreover, a triple therapy in pre-clinical murine models consisting of T-VEC and two other immunotherapies resulted in an astounding 80% complete tumor eradication (Kalsi et al., 2024). Other clinically used OVs include the adenovirus H101 (Oncorine) for head and neck cancer in China, in addition to the conditionally approved HSV-1 Teserpaturev (Delytact) for malignant glioblastoma in Japan (Muthukutty and Yoo, 2023). However, such robust OVT performance remains limited to some tumors and conditions where viral spread and the targeted activation of an immune response are achievable. Even if OVs do reach tumors through intravenous administration for deep or metastasized cancers, the profoundly immunosuppressive nature of solid tumors is likely to hinder OVT responses. Dense biological barriers, low antigenicity, and other factors greatly reduce the immune system’s capability to activate the long-term immune response that distinguishes OVT. It is therefore critical to understand how the TME produces such immunosuppression to address OVT’s challenges.

## 3 Barriers of the tumor microenvironment

Despite OVs’ immunogenic nature, their overall therapeutic effectiveness is greatly reduced by the combination of immunosuppressive factors in the TME. In solid tumors, the TME’s immunosuppressive nature hinders viral and immune cell infiltration, drug effectiveness, and immune activation through a combination of physical, cellular, and molecular barriers (Kim and Cho, 2022; Wang et al., 2025). This is characteristic of a ‘cold’ tumor, an immunophenotype used to describe tumors that are generally unresponsive to immunotherapies (Figure 2). In contrast, hot tumors are characterized by increased inflammation, immune cell infiltration, and immune checkpoint expression. As a result, hot tumors are much more responsive to immunotherapies such as OVT and the commonly co-administered immune checkpoint inhibitors (ICIs) (Wu et al., 2024).

**Figure 2:**
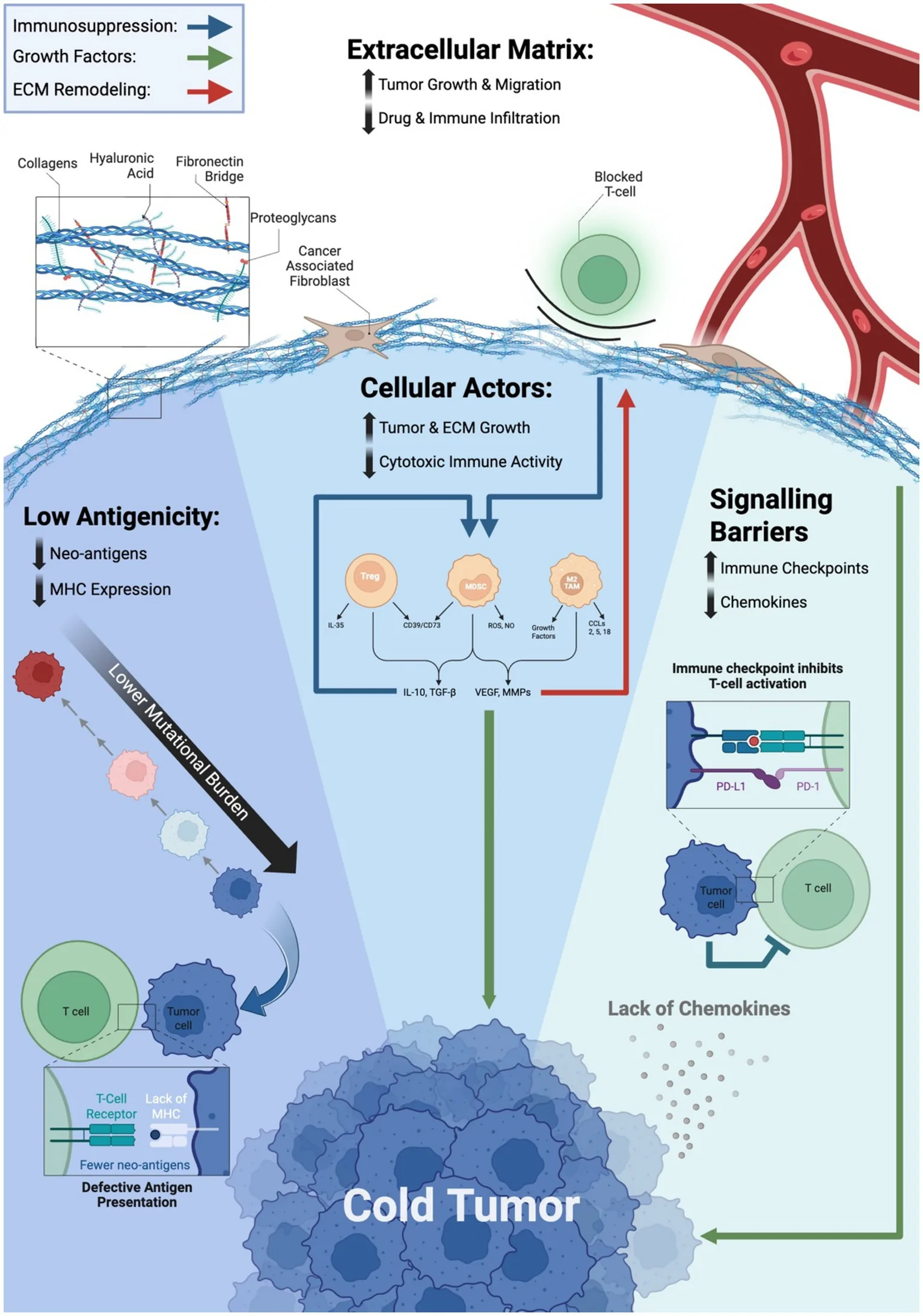
A simplified illustration of the various immunosuppressive factors at play in the tumor microenvironment of cold tumors. Created in BioRender. Boulfrad (2026). https://BioRender.com/fizmhju.

### 3.1 Physical barriers: the extracellular matrix

The first formidable barrier for the immune system is the desmoplastic stroma that surrounds solid tumors. As a dense extracellular matrix (ECM), it consists of a plethora of components, including a highly cross-linked arrangement of fibrillar collagen types I, II, III, IV, and V; water-attracting hyaluronic acid (HA), leading to elevated interstitial fluid pressure and compressed blood vessels; as well as cell growth and angiogenesis-promoting proteoglycans (Lee et al., 2025). The ECM therefore limits the movement of OVs, therapeutic drugs, and immune cells. Moreover, the desmoplastic stroma contains cellular actors, such as Cancer-Associated Fibroblasts (CAFs), which secrete growth factors and immunosuppressive cytokines, therefore exacerbating desmoplasia and even inducing resistance to immunotherapies (Okuyama et al., 2025). When combined with the ECM’s stiffness, this dysregulates biomechanical and chemical signaling pathways such as YAP/TAZ, therefore promoting tumor cell growth and survival (Fu et al., 2024; Chitty and Cox, 2025). In many cases, metastasis is facilitated by the ECM as it provides a scaffold for tumor cell migration (Chitty and Cox, 2025). The ECM is an active member of the TME with many mechanisms at play, therefore rendering it a key target for enhancing immunotherapies.

A novel advancement in OVT targets the ECM through the degradation of HA. Oncolytic vaccinia virus (OVV) was constructed to encode the human-derived Hyaluronidase-1 to create the ECM-degrading OVV-Hyal1. When tested on mouse subcutaneous tumor models, significant improvements in viral spread, immune cell infiltration and activation, and chemotherapeutic drug penetration were reported (Wang, S. et al., 2024). The Warburg effect, cancer cells’ constant lactic acid fermentation, lowers TME pH and facilitates the action of the Hyal1 enzyme (De Leon-Oliva et al., 2025; Jin et al., 2026). However, HYAL1’s therapeutic potential is plagued by uncertainty regarding its role in cancer progression. In fact, the enzyme has been demonstrated to actively contribute to tumor progression in cases such as male genitourinary cancers and aggressive laryngeal carcinoma (Jin et al., 2026). It has also been found to facilitate the brain metastasis of triple-negative breast cancer (Hamester et al., 2022). It is therefore imperative that the context-dependent effects of ECM degradation are better understood to safely address this challenge.

### 3.2 Low antigenicity

One of the main reasons for the low antigenicity of cold tumors is defective antigen presentation machinery (APM), which allows tumor cells to “hide” their antigens from immune cells. This can occur when tumor cells downregulate or completely lose the expression of their major histocompatibility complexes (MHC) classes I and II, which are crucial for the activation of CD8+ cytotoxic T-cells and CD4+ helper T-cells, respectively (Taylor and Balko, 2022; Wang, J. et al., 2024). As a result of defective APM, cytotoxic T-cell activation against cancer cells is impaired. Many underlying mechanisms can result in this phenotype, including somatic mutations, epigenetic changes, reduced mRNA stability, increased protein degradation, and disruption of endocytic trafficking (Kong et al., 2025).

Another key driver of low tumor antigenicity is low Tumor Mutational Burden (TMB), or fewer somatic genetic mutations. Lower TMB yields fewer neoantigens that can be recognized as foreign by immune T-cells (Doig et al., 2022). Generally, higher TMB corresponds with improved responses to immunotherapies (Budczies et al., 2024).

Attempts to address low tumor antigenicity include the previously discussed incorporation of immunostimulatory GM-CSF cytokine into T-VEC, as well as the removal of viral genes that downregulate the expression of MHC-I in tumor cells (Russell and Barber, 2018). However, this remains a formidable challenge for OVT, which heavily relies on the immune system’s ability to recognize cancerous cells to bring long-term adaptive immune responses to fruition. An inability to do so greatly reduces OVT’s therapeutic effectiveness and therefore prevents it from realizing its full clinical potential. Addressing this challenge would both increase OVT’s accessibility to a broader range of heterogeneous tumor types and increase the effectiveness of co-administered immunotherapies.

### 3.3 Immunosuppressive cellular actors

Immunosuppression in the TME is largely carried out by host cells that are ‘re-educated’ and become tumor-associated (Park et al., 2022). For example, Regulatory T-Cells (Tregs) secrete immunosuppressive and anti-inflammatory cytokines including IL-10 and TGF-β (Li et al., 2020; Zhang et al., 2024). Strategies being explored to target Treg cells include selective immune checkpoint blockade and co-stimulation of T-cells through bispecific antibodies (Sainson et al., 2018). However, Treg cells are essential for systemic immune tolerance and share many signaling pathways with other T-cells, increasing the difficulty of targeting Tregs in the TME without off-target depletion of T-cells or affecting body-wide immune tolerance (Kvarnhammar et al., 2019).

Moreover, Tumor-Associated Macrophages (TAMs) are polarized macrophages that are broadly categorized into the M1 tumor-suppressing and M2 tumor-supporting phenotypes. M2 TAMs are polarized by immunosuppressive stimuli including TGF-β, IL-4, IL-10, and IL-13 (Hourani et al., 2021). They support tumor growth, angiogenesis, and metastasis, while also producing immunosuppressive cytokines (Park et al., 2022; Kzhyshkowska et al., 2024). Attempts to mitigate M2 TAMs’ effects on the TME include inhibiting the activation of M2 phenotypes by targeting CSF-1R and reprogramming M2 TAMs to the M1 phenotype through PI3Kγ inhibition.

Myeloid-Derived Suppressor Cells (MDSCs) are a heterogeneous group of immature myeloid cells that promote angiogenesis, metastasis, and the development of other immunosuppressive cell types. MDSCs’ inherent heterogeneity severely complicates safely targeting and/or neutralizing them (Lasser et al., 2024). However, some STAT3/TKI inhibitors such as Galiellalactone and sunitinib have demonstrated promising results in addressing MDSCs.

Collectively, current therapeutic strategies targeting immunosuppressive cellular actors—including Treg depletion, TAM repolarization, and MDSC inhibition—have achieved partial preclinical success but generally remain constrained by off-target side effects, systemic toxicity, and limited effectiveness as monotherapies. There is therefore a need for novel, precise, and dynamic therapeutic platforms.

### 3.4 Chemical and signaling barriers

A key characteristic of cold tumors is the lack of pro-inflammatory cytokines and chemokines, which are crucial for attracting immune cells to the tumor site. For example, the lack of CXCL9, CXCL10, and CXCL11 chemokines contributes to immune exclusion from the tumor (Karin, 2021; Ouyang et al., 2024). Nonetheless, the release of DAMPs, and the resulting production of chemokines during OVT help bring the tumor onto the immune system’s “radar.” Moreover, many cancers overexpress co-inhibitory immune checkpoints, which bind to immune checkpoints on immune cells to inhibit cytotoxic responses. This halts the immune response of the few T-cells that do manage to infiltrate the TME (Meng et al., 2024; Ouyang et al., 2024). Some closely studied immune checkpoints include the programmed cell death protein 1 (PD-1) receptor and Cytotoxic T-Lymphocyte-Associated Protein 4 (CTLA-4) (Meng et al., 2024).

A promising solution for immune checkpoint activation is immune checkpoint inhibitors (ICIs) (Meng et al., 2024; Ouyang et al., 2024). These effectively allow immune cells to fulfill their cytotoxic functions in the TME. However, because this immunotherapy systemically inhibits immune self-tolerance, autoimmune-like symptoms throughout the body are a common concern (Bajwa et al., 2019). Moreover, the TME tends to gain resistance against specific ICIs over time. Again, a precise and dynamic therapeutic platform is much needed.

## 4 Bacteriophages: bridging the gap

Bacteriophages, also known as phages, are a diverse group of bacterial viruses and are non-pathogenic to humans. They are abundant on human surfaces in direct contact with the environment, such as the skin, oral cavity, lungs, gut, and urinary tract (Van Belleghem et al., 2018; Panneerselvam et al., 2025). These highly biophysically stable, and therefore portable, viruses have recently resurged as promising tools in addressing antimicrobial resistance and targeting oncogenic bacteria through phage therapy, a treatment used in Russia, Georgia, and Poland for over a century (Gordillo Altamirano and Barr, 2019; Ireland, 2023; Moon et al., 2025). However, the potential for phages as a therapeutic platform in cancer extends beyond directly targeting bacteria. OVs could greatly benefit from the transferrable mechanisms regulating phages’ immunogenicity, high specificity, genetic manipulability, ability to penetrate biological barriers, and delivery of large genetic and therapeutic payloads (Panneerselvam et al., 2025; Ramadan et al., 2025). In this context, a few key features of phages are most prominent (Figure 3).

**Figure 3:**
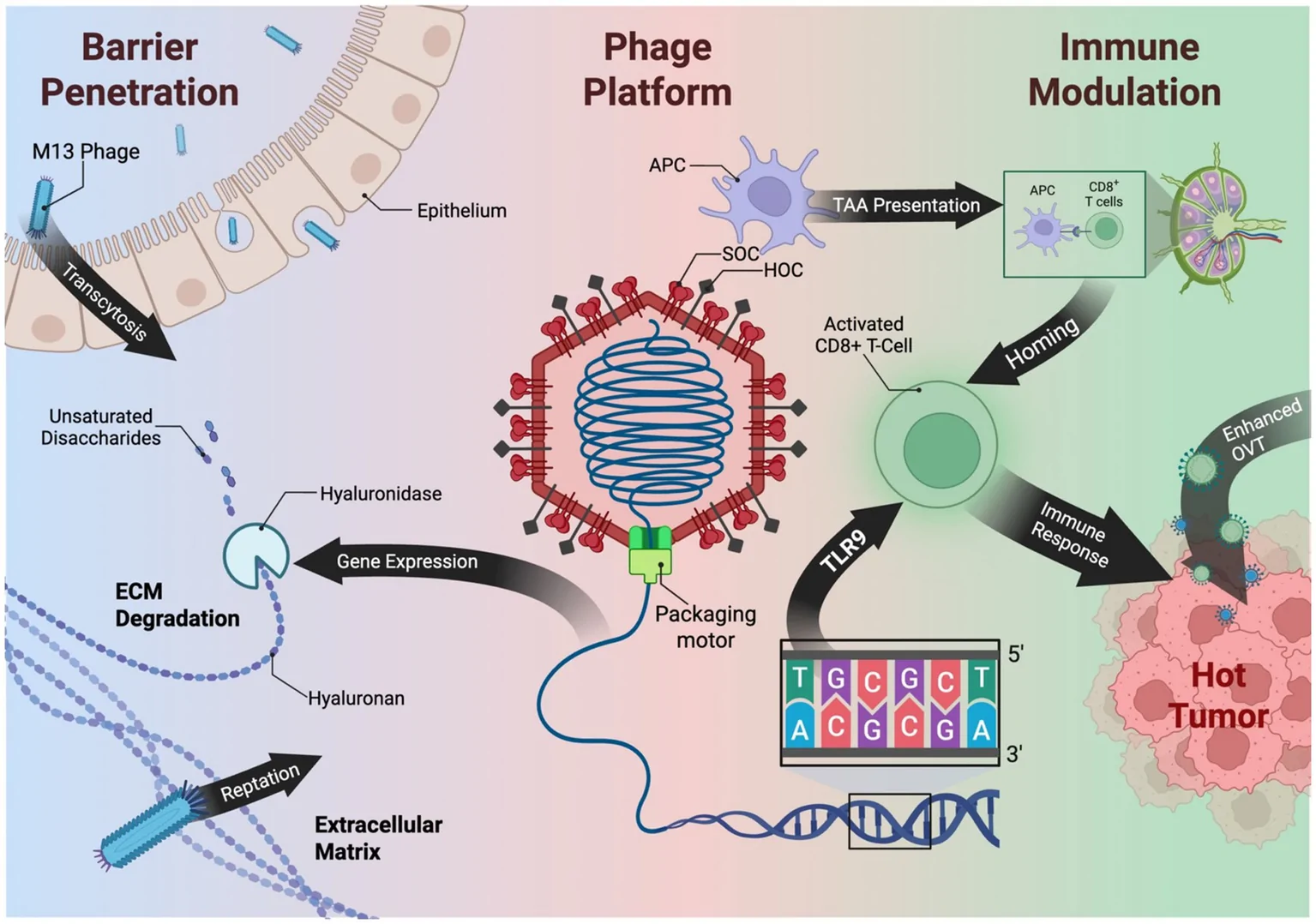
An illustration of the various phage mechanisms enhancing OVT delivery and anti-tumor immunity. Created in BioRender. Boulfrad (2026). https://BioRender.com/zan72xz.

### 4.1 Barrier breaching mechanisms: navigating dense environments

Phages have evolved sophisticated morphological and enzymatic mechanisms to bypass complex biological barriers. Although these systems are largely optimized for the penetration of bacterial biofilms, matrices, and capsules, the transfer of their functionality has the potential to improve OV spread, infiltration, and controlled degradation of the extracellular matrix.

Phages’ remarkable ability to cross biological and endothelial barriers is largely due to effective transcytosis mechanisms. Following receptor-mediated endocytosis, bacteriophages can be transported from one pole of a cell through a vesicle to be released at the opposite pole. Through this method, phages such as T4, T5, T7, SP01, SPP1, and P22 can easily cross through several cell layers, thus allowing for their transport across epithelial tissues of the intestines, lungs, liver, kidneys, and brain (Urban-Chmiel and Pyzik, 2025).

Moreover, the filamentous structure of certain phages, namely M13, allows for much higher translocation rates. This is likely due to the ability of filamentous phages to penetrate epithelial and endothelial cells through a variety of methods, including macropinocytosis, clathrin-mediated endocytosis, or caveolae-mediated endocytosis (Urban-Chmiel and Pyzik, 2025). In the context of the TME, such a high aspect ratio allows filamentous viruses to undergo reptation, a snake-like motion that enables diffusion through pores in dense matrices akin to the tumor ECM.

Phages also employ an arsenal of enzymes to degrade bacterial biofilms and capsules. Two major classes of enzymes are hydrolases and lyases, and the difference between the two is paramount for the TME. While hydrolases break bonds through hydrolysis, lyases do so without the use of water as a substrate (Morisseau, 2022). They instead use elimination to form multiple bonds or rings (Manubolu et al., 2018). The previously discussed human-derived hyaluronidase HYAL1 is a hydrolase enzyme. HYAL1 cleaves 1,4-β-glycosidic linkages to form saturated disaccharides (Reitinger et al., 2009). These low molecular weight HA (LMW-HA) fragments have been found to bind to Toll-like receptors (TLR2/4) and CD44 to activate the predominantly tumor-supporting NF-κB and MyD88 signaling pathways, thus leading to increased long-term inflammation and invasion (Bourguignon et al., 2011; Carvalho et al., 2023; Lu et al., 2025). In contrast, the phage-derived hyaluronate lyase (HylP) breaks 1,4-glycosidic bonds in hyaluronan through β-elimination. The resulting unsaturated disaccharides (ΔHA2) do not contribute to pro-inflammatory or tumorigenic pathways. In fact, ΔHA2 has been demonstrated to inhibit lipopolysaccharide-induced inflammation (Han et al., 2022). Moreover, bacterially derived and encoded hyaluronidases have already demonstrated effectiveness in combating various tumor types (Lee et al., 2010; Ebelt et al., 2020; Thirumurthy et al., 2023; Ebraheem et al., 2024).

### 4.2 Payload capacity

A significant barrier in OVT is the limited accommodation for transgene insertions or restrictive genomic capacity of mammalian viral vectors. This is exemplified by the gold standard of gene delivery—Adeno-Associated Viruses (AAVs)—which can efficiently deliver only ~4.7 kb of genetic information (Wu, Z. et al., 2010). Although there are larger OVs such as the 150 kb herpesvirus and the 190 kb vaccinia virus, their therapeutic applications are generally limited due to inherent pathogenicity or cytotoxicity (Manservigi, 2010; Smith et al., 2013; Lin et al., 2023).

Nonetheless, large-genome bacteriophages, namely the ~170 kb T4 phage, offer a unique solution through their ‘head-full’ DNA packaging mechanisms (Rao et al., 2023). During assembly, DNA is driven into the phage capsid through an ATP-powered motor until the capsid reaches its full physical capacity (Rao et al., 2023; Rao, 2025). This allows the T4 phage to dedicate its entire capsid to large, complementary therapeutic circuits, an ability further strengthened by the removal of phage genes. Moreover, the high pressures inside the tightly packed capsid, up to 60 atmospheres, transition the DNA into a highly dense, biophysically stable liquid-crystalline state (Rao, 2025). This renders the genetic payloads resistant to environmental denaturation and enzymatic degradation through nucleases. These mechanisms drive phages’ ability to act as robust ‘Trojan Horses’ capable of reliably delivering large therapeutic payloads, opening the door for the mitigation of each TME’s specific challenge (Karimi et al., 2016).

### 4.3 Phage display

Bacteriophages also offer a platform for the high-valency surface display of peptides in a repetitive and structured manner, referred to as phage display. This is achieved through the fusion, at the genetic level, of target polypeptides to both highly dense major coat proteins such as pVIII, with 2,700 copies on M13 phages, and minor coat proteins such as pIII, which, despite a low copy number of five, can accommodate large polypeptide fusions. Conveniently, an overlap is found in the Highly Antigenic Outer Capsid (HOC) and the Smaller Outer Capsid (SOC) proteins. T4 phages can be decorated by 155 and 810 of these coat proteins, respectively while also displaying significantly larger molecules (Qin et al., 2010; Gamkrelidze and Dąbrowska, 2014; Fokine et al., 2023). Although the research in this technology is predominantly focused on the screening of diverse antibody libraries for binding to specific targets, it does have potential for therapeutic applications (Frei and Lai, 2016). The high-avidity presentation of molecules with affinity for specific integrins allows phages to precisely and reliably bind to target cells. This is exemplified in the display of the RGD-4C ligand, which can recognize α_v_β_3_ and α_v_β_5_ integrins on cancer cells and tumor-associated neo-vasculature (Chongchai et al., 2024; Ooi and Yeh, 2024). Furthermore, the peptide display of tumor-associated antigens has a strong potential to initiate or amplify existing adaptive immune responses against tumor cells (Dong et al., 2022). This occurs through cross-presentation with APCs reliably triggered by structured and repetitive antigen presentation, effectively creating anti-tumor immunity as a by-product of anti-viral immune responses. However, the therapeutic success of TAA-display strategies is contingent on the proper functioning of antigen presentation machinery in tumor cells to effectively prime specialized T-cells.

### 4.4 Immunomodulation

When aligned with systemic TAA presentation or tumor-specific OVT, the introduction of phage-derived immunomodulatory mechanisms can assist in fine-tuning the complex TME to benefit from desired anti-tumor immune responses while limiting pro-tumor and angiogenic chronic inflammation. While many of the underlying mechanisms are yet to be discovered, phages have been demonstrated to induce both immunogenic and anti-inflammatory responses in eukaryotic environments.

For example, unmethylated CpG dinucleotide motifs inherent in phage DNA act as potent immunostimulators when phagocytosed by APCs through the activation of Toll-like receptor 9 (TLR9) (Golkar and Jamil, 2012). Optimizing the incorporation of CpG motifs into chimeric vectors for the desired immune responses can be greatly facilitated by the novel Bacteriophage Risk Index (BRI), which reliably quantifies phage immunogenic potential based on dinucleotide frequency, motif spacing, and sequence context (Kharrat et al., 2025).

On the other hand, phage capsid proteins have been demonstrated to induce anti-inflammatory effects by inhibiting the NF-κB signaling pathway, thus resulting in the reduced expression of key pro-tumor chronic inflammatory cytokines, namely IL-1β, IL-6, and, in some cases, TNF-α (Urban-Chmiel and Pyzik, 2025). Phages also employ further mechanisms, such as limiting the overproduction of reactive oxygen species (ROS), inhibiting the activation of Toll-like receptor 4 (TLR4), and various other mechanisms (Cafora et al., 2021). Overall, the immunomodulatory capabilities of phages remain an ongoing area of research, with the elucidation of underlying mechanisms unlocking the translation of precise immune modulation in immunotherapies like OVT.

## 5 The phage-OV hybrid: a synergistic approach

There are essentially two main ways to reap the benefits of phage-derived systems for OVT and cancer therapeutics in general. The first entails the creation of a hybrid viral vector by merging the genome of an OV or associated mammalian viral vector into a phage backbone or capsid. The second, discussed later, is the modular incorporation of phage-derived or phage-inspired mechanisms and systems into replication-capable OVs at the genetic level (Figure 4).

**Figure 4:**
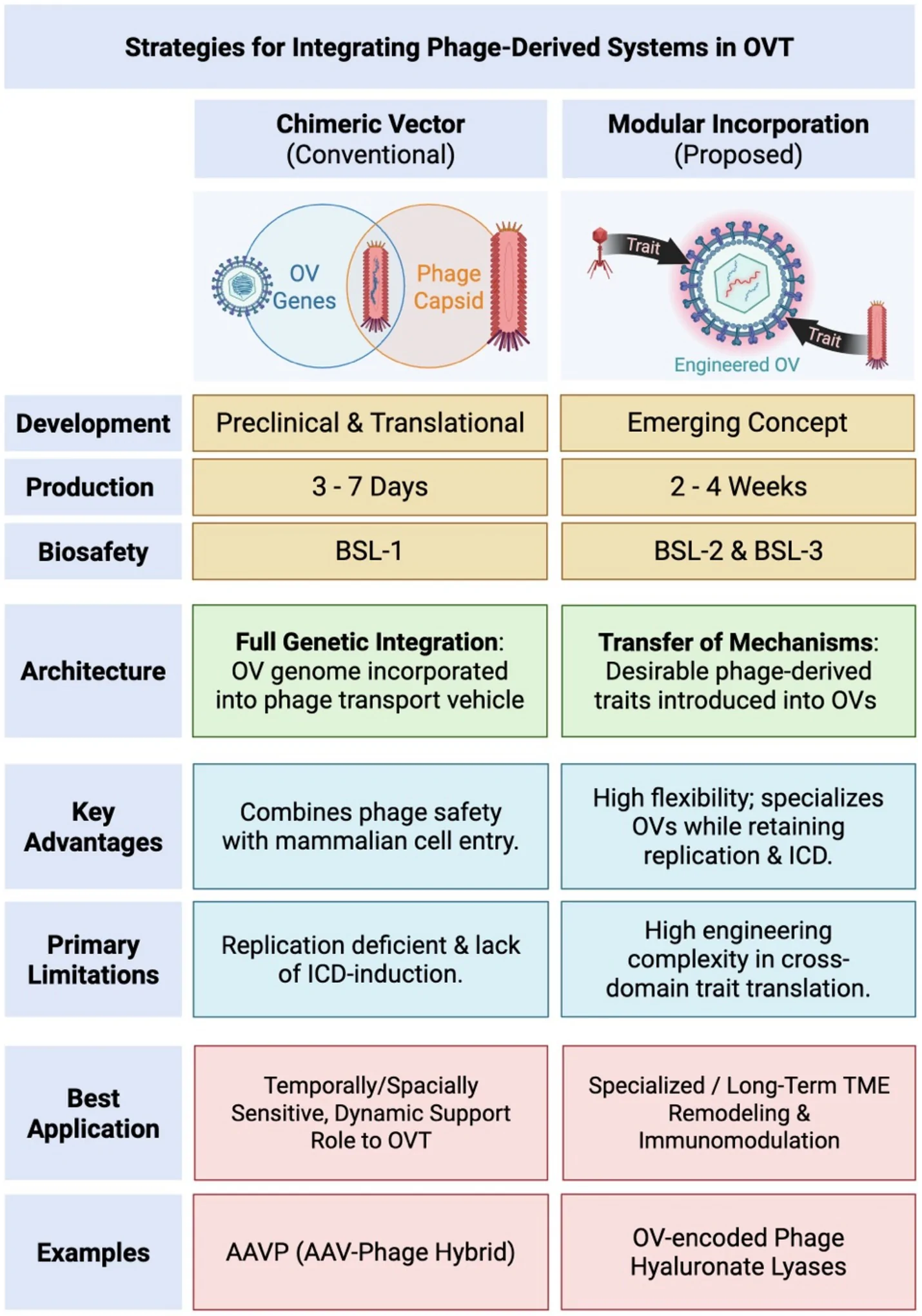
Comparative framework of phage-OV integration strategies. Established Chimeric Hybrids (left) focus on structural fusion, creating robust viral vectors. The proposed Modular Incorporation (right) aims to specialize OVs to modulate the TME through the phenotypic transfer of desirable phage traits. Created in BioRender. Boulfrad (2026). https://BioRender.com/k65s4hw.

### 5.1 Chimeric vectors

The creation of bacteriophage-OV viral vectors much more holistically captures the safety, manipulability, and ease of production of phages through chimeric hybridization. It has promise as an effective method to precisely deliver significant therapeutic payloads to targeted tumors through stable vectors, penetrate biological barriers, and activate adaptive immune responses through the phage display of TAAs specific to the tumor of interest (Zhang and Wang, 2024). Nonetheless, this method does bypass and therefore loses the benefits of viral replication and lysis of cancer cells. Although delivery of suicide genes and the induction of tumor cell apoptosis have been achieved by such chimeras, the high immunogenicity of tumor cell lysis and the resulting release of DAMPs and TAAs is not matched (Smith et al., 2016; Sittiju et al., 2024). Moreover, the longevity of these viral vectors in the TME is greatly reduced by their replication deficiency. This is further exacerbated by the inherent immunogenicity and rapid clearance of phages by immune cells (Table 1).

**Table 1:** Comparative table of experimental phage-OV and hybrid systems to date.

Treatment	Study type	Function	Mechanism	Advantages	Challenges/next steps	Reference
AAVP (RGD-4C)	*In vivo* (murine)	First hybrid AAVP vector.	Cis elements of AAV integrated into filamentous phage (M13) capsid. Genetic payload driven by CMV promoter and flanked by AAV2 ITRs.	No liver-detargeting, functions in the presence of anti-phage antibodies. AAV ITRs enhanced vector persistence.	Low vector efficiency, achieving Good Manufacturing Practice, induction of humoral immune responses	Hajitou et al. (2006)
AAVP (RGD-4C)	*In vitro*	Improving vector transduction via disruption of endolysosomal pathway	Co-administration of acidic organelle neutralizer chloroquine	Dose and cell-type dependent improvements in vector efficiency and transduction capabilities. AAVP mediated gene expression increased by a factor of 2.8–9.2 at 4 h with a 200 μM chloroquine treatment.	The high concentrations of chloroquine required are highly cytotoxic to healthy cells.	Stoneham et al. (2012)
AAVP (RGD-4C)	*In vitro* and *in vivo* (murine)	Improving hybrid transgene expression	Co-administration of proteasome inhibitors MG132 and LLnL	Longer vector presence. Enhanced tumor-specific transgene expression	Dose and cell-type dependent cytotoxicity.	Przystal et al. (2013)
AAVP (RGD-4C)	*In vitro*	Improving AAVP transduction	A system of naturally negatively charged AAVP and a synthetic cationic polymer creates positively charged phage aggregates.	Improved specificity in binding, gene-expression, and killing of cancer cells. Improved transduction efficiency.	More preclinical studies to assess phage/polymer systems *in vivo* are necessary.	Yata et al. (2014)
AAVP (RGD-4C)	*In vitro* and *in vivo* (murine)	Improving AAVP transport through ECM degradation	Co-administration of antihypertensive losartan to inhibit collagen I *de novo* synthesis.	Preferentially impacts metabolically active tumor cells while reducing metastatic side-effects	Due to the effectiveness of ECM modulation being highly dependent on dose and cell-type, careful testing needs to be carried out before generalizing this approach.	Yata et al. (2015)
AAVP (RGD-4C)	*In vitro* and *in vivo* (murine)	Improving hybrid transgene expression	Co-administration of genistein, found to inhibit proteasomal degradation, enlarge nuclear pores, and promote conversion of ssAAVP DNA into more stable and transcriptionally active dsAAVP DNA.	Longer vector presence. Reduced cytotoxicity (effective doses are below cytotoxic half-maximal inhibitory concentrations IC50)	Genistein can have tumor-supporting and tumor-degrading properties depending on dosage and factors that are not yet fully known.	Tsafa et al. (2016)
AAVP (RGD-4C)	*In vitro* and *in vivo* (murine)	Improving AAVP transduction	Combination of AAVP with inherently bio-compatible gold (Au) nanoparticles to create a “transducing matrix.”	Improved transduction capabilities, potential to track AAVP complexes through X-ray based imaging without changing payload, and potential to leverage magnetic properties for spatially precise or patterned delivery	A highly detailed and intricate understanding of the TME and how its components interact is necessary to appropriately leverage this technology’s temporal and spatial precision.	Smith et al. (2017)
AAVP (RGD-4C)	*In vitro*	Improving hybrid transgene expression	Co-administration of HDAC6 specific inhibitor C1A	Specifically inhibits HDAC6, and not general HDAC, suggesting a significant boost in AAVP efficiency in humans with much lower toxicity and cost	*In vivo* assessments of this promising technique across multiple cancer types are necessary to generalize these findings.	Campbell et al. (2018)
RGD4C-AKAS-AAVP	*In vitro* and *in vivo* (murine)	Mitigating pre- and post-internalization challenges	Displaying RGD4C on pIII minor coat protein, short-charged neutralizing peptide AKAS on multi-thousand copy pVIII coat protein, and histidylated fusogenic peptide H5WYG with endosomal buffering capacity.	Results in a trifunctional AAVP vector with zwitterionic properties, interacted less with fibrinogen and antiphage antibodies, improved endosomal escape, significantly higher transgene expression	Creating a tetrafunctional AAVP would allow for the simultaenous display of AKAS and H5WYG.	Suwan et al. (2019)
Chemovirotherapy (AAVP x doxorubicin)	*In vitro*	Overcoming cancer resistance	Co-administration of RGD4C/AAVP-HSVtk/GCV targeted suicide gene therapy and doxorubicin chemotherapy	Increased transgene expression, synergistic effect to destroy both human and murine tumor cells in 2D and 3D spheroid settings.	When assessing the same combination *in vivo*, hurdles such as systemic side effects and developed resistance need to be mitigated.	Tsafa et al. (2020)
MN administered PeptiCRAd vaccine	*In vivo* (murine)	Safely inducing tumor-specific immune response	VALO-mD901 adenovirus with the E3 region replaced by human CMV promoter and murine CD40L and OX40L genes coated with poly-K murine SIINFEKL peptides.	Pre-vaccinated mice demonstrated complete tumor rejection. Increased cDC1 population in lymph nodes and increased effector memory T cells in body.	Scalability of MN production, long term stability of MNs, and stability of MN vaccines after storage is yet to be explored.	D’Amico et al. (2025)
PeptiCRAd-1 combination therapy human trial	*In vivo* (human)	Safely inducing tumor-specific immune response	CD40L and OX40L expressing adenovirus electrostatically coated with TAAs NY-ESO-1 and MAGE-A3, administered alongside pembrolizumab ICI and after low-dose cyclophosphamide. Various solid tumor types expressing the corresponding TAAs were included.	No dose-limiting toxicities, treatment-related serious adverse events, or unexpected safety signals reported. Favorable safety profile, and induction of systemic and intratumoral immune responses against advanced tumors.	Small sample size of 7 patients, more studies needed to replicate findings across solid tumor types.	Kuryk et al. (2026)
Hyaluronidase Lyase	*In vitro*	Safely degrade HA in the ECM	Comparing the effects of prokaryotic HyIP and mammalian BTH hyaluronidases on various breast cancer cell lines.	HyIP’s metabolic process produced non-detrimental oligosaccharides that, by disrupting Erk1/2 activation and RhoA expression, reduced breast cancer cell proliferation, migration, and invasion	Only tested *in vitro*. *In vivo* assessments of the a potential immune response against prokaryotic hyases are needed.	Lee et al. (2010)
Hyaluronidase Lyase	*In vitro*	Safely degrade HA in the ECM	Hyase derived from Brucella intermedia MEFS (OR794010) was purified and tested on various cancerous and healthy cell lines.	Strong anticancer effects on colon, lung, skin, and breast cancer cell lines with low toxicity against healthy somatic cells. A DPPH scavenging activity of 65.49% and total antioxidant activity of 71.84% were recorded at a hyase concentration of 200 μg/mL	Only tested *in vitro*. *In vivo* assessments of the a potential immune response against prokaryotic hyases are needed.	Ebraheem et al. (2024)

AAVP = adeno-associated virus-phage; AKAS = Ala-Lys-Ala-Ser short-charged neutralizing peptide; BTH = bovine testes hyaluronidase; CD40L = CD40 ligand; CMV = cytomegalovirus; cDC1 = type 1 conventional dendritic cells; DPPH = 2,2-diphenyl-1-picrylhydrazyl; Erk1/2 = extracellular signal-regulated kinase 1 and 2; GCV = ganciclovir; HDAC6 = histone deacetylase 6; HSVtk = herpes simplex virus thymidine kinase; IC_50_ = half-maximal inhibitory concentration; ITR = inverted terminal repeat; MN = microneedle; MAGE-A3 = melanoma-associated antigen A3; NY-ESO-1 = New York esophageal squamous cell carcinoma 1; OX40L = OX40 ligand; RhoA = Ras homolog family member A; RGD = Arg-Gly-Asp peptide motif. Information in this table was synthesized from experimental studies and adapted from comprehensive reviews (Fakhiri and Grimm, 2021; Islam et al., 2026).

Nonetheless, this transient and precisely controllable nature could be beneficial in certain temporally sensitive therapeutic applications where prolonged activity is undesirable. For example, the matrix metalloproteinase family (MMP-1–23) helps tumors in ECM remodeling, supporting tumor growth through changes in ECM composition and stiffness (Roy et al., 2009). Broad-spectrum MMP-targeting therapeutics had to be halted due to challenges in toxicity and disease acceleration (Sleeboom et al., 2024). Different members of the MMP family may have different roles in ECM regulation, and the effects of targeting them are highly dependent on the cancer stage. In fact, some MMPs such as MMP-9 have been shown to promote tumor progression in early cancer stages but restrict malignant growth in later stages (Coussens et al., 2000). However, there is potential in targeted MMP inhibitors, especially when applied to pre-metastatic tumors. Non-replicating phage-OV chimeras are especially suited for the delivery of this type of therapeutic payload, as its applications are both temporally and spatially sensitive.

Furthermore, the immunogenic nature of these recombinant viruses allows the immune system to rapidly initiate an adaptive response against the TAAs displayed on phage coat proteins. Establishing such targeted yet systemic responses early on can condition the immune system against the tumor of interest prior to the administration of lytic OVs. This could further sensitize the patient’s immune response to upcoming OVT, help reduce the risk of metastasis, and improve overall prognosis.

By far the best-studied example of mechanistic phage-OV synergy is the Adeno-Associated Virus/Phage (AAVP) hybrid system first developed by Hajitou et al. (2006). This vector merged the mammalian-optimized transduction capabilities of AAV vectors with the stability and precision of bacteriophages. The AAV genome and its cis-acting elements were integrated, along with inverted terminal repeats (ITRs) for improved and prolonged transgene expression, into the filamentous M13-derived bacteriophage. This phage backbone uses peptide display of the RGD4C ligand to bind to the αvβ3 and αvβ5 integrins, cell-surface receptors prevalent on tumor vasculature and across various cancer types (Yang Zhou et al., 2020). This phage-OV chimera enabled ligand-dependent transduction of tumor tissue with significant liver de-targeting, effectively addressing a major limitation faced by other viral vectors. Subsequent development of this platform focused on enhancing transduction efficiency by improving endosomal escape, reducing proteasomal degradation, and ECM penetration (Table 1). Applications and variations of AAVP have been able to accurately induce apoptosis (Smith et al., 2016; Sittiju et al., 2024), reduce tumor metabolism and hypoxia (Smith et al., 2016), deliver CRISPR-Cas9 to directly introduce tumor-suppressing genes (Yang Zhou et al., 2020), and increase susceptibility to drugs (Przystal et al., 2019). Therefore, although chimeric vectors such as AAVP are replication-deficient, their superior payload delivery capabilities render them highly promising therapeutic platforms for tumor modulation, especially when administered in coordination with lytic OVs.

### 5.2 The modular incorporation

On the other hand, unlike phage-OV chimeric vectors that merge entire genomes or capsids, the modular incorporation of bacteriophage-derived components into OVs theoretically maintains the replication-capable and ICD-inducing nature of lytic OVT while also benefiting from the translation of the immunogenicity, specificity, and barrier penetration capabilities of phages. This modular synergy provides a flexible framework for addressing OVT’s challenges in the TME, particularly when simultaneously targeting multiple sources of immunosuppression. Another benefit of phage-derived modular incorporation is the vast diversity of phage mechanisms, presenting a largely untapped pool of potentially transferable immunogenic systems. These can be continually researched to iterate on future modularly synergized OVs.

However, a drawback of this framework is the inability to holistically transfer phages’ ease of production and genetic manipulability. A further-reaching implication is the difficulty of introducing a large set of phage-derived mechanisms into OVs without compromising OV stability and replication efficiency. Moreover, directly introducing phage-derived genes of interest into OVs is not feasible due to fundamental differences in genetic replication and expression machinery between prokaryotic and mammalian viruses. Factors that contribute to this disparity include systemic differences in the presence or use of codon preferences, polyadenylation signals, ribosomes, introns, and exons (Cooper, 2000; Kanhere, 2005; Botzman and Margalit, 2011; Zhou et al., 2018).

Nonetheless, these challenges can be addressed by changing how recombinant phage-OVT is approached. Instead of working toward a ‘universal OV’ that combats all the TME’s complex immunosuppressive systems, creating a set of specialized OVs with fewer but more relevant modifications for targeting specific aspects of the TME would raise their combined therapeutic potential while maintaining replication efficiency and viral stability. As for incompatibility with direct genetic transfer, a solution likely lies in phenotypic translation, replicating the function of phage-derived mechanisms through synthetic redesign or genetic engineering to achieve cross-domain mimicry. Practically, this could lean toward the introduction of phage-inspired mechanisms into specialized replication-capable OVs.

A concrete example of how the modular transfer of phage-inspired mechanisms into OVs can be beneficial is seen in the induction of anti-tumor immune responses through tumor associated antigens. OVs have been genetically engineered to encode TAAs or present them on coat proteins. This allows for the overexpression of antigens in infected cells resulting in the prolonged and high presence of TAAs in the TME. This was shown to prime T-cells against the antigens of interest and potentially render OVs beneficial as a combination therapy with ICIs where they would otherwise be detrimental (Xiao et al., 2026). Nonetheless, it is not feasible to translate this concept into therapeutic applications due to the slow and expensive process of genetically engineering personalized OVs based on the antigens present at the tumor of interest, especially when compared to clinical timelines. Moreover, the highly heterogeneous and dynamic nature of the TME could necessitate the priming of T-cells against different or new antigens over time, a demand that antigen-encoding OVs are unlikely to accommodate. This is where phage-inspired transfer comes into play. Phage display’s temporal specificity and ability to modularly replace the displayed peptide without altering the underlying mechanisms has been mirrored into OVs through the development of PeptiCRAd. This platform allows for the external decoration of fixed OV capsids with selected peptides through the non-covalent coating of negatively charged oncolytic adenoviruses and vaccinia virus capsids with TAA-presenting MHC-I epitopes extending from poly-lysine tails through electrostatic interactions (Feola et al., 2022). This system allows a simple 15-min incubation step to coat the replication-capable OV with an external epitope layer (Ylösmäki et al., 2021). PeptiCRAd not only offers feasible and rapid personalization, but it is also competitive with antigen-encoding OVs in anti-tumor T-cell priming. In fact, cross-presentation experiments demonstrate that PeptiCRAd yields faster presentation and has a more immunogenic profile (Kuryk, 2026). Moreover, it overcomes the immunological shield: a major issue with OVs is that the immune system primarily attacks the virus, ignoring the tumor. PeptiCRAd acts like a Trojan horse—by coating the capsid in tumor peptides, it redirects the immune response away from the virus and directly toward the cancer cells. However, unlike antigen-encoding OVs, PeptiCRAd coating is not genetically amplified with replication. Rather, it is a first-dose phenomenon that ends while the OV continues to proliferate. Nonetheless, much like phage-OV chimeric hybrids, the temporal specificity of this platform could be a strength when the dynamic nature of the TME requires T-cell priming to be redirected to a new set of antigens. Therefore, PeptiCRAd serves as a rapid, modular approach that could be co-administered along with the persisting and amplifying antigen-encoding OVs to achieve controlled, highly specific immunogenicity. Other methods exist, such as biomimetic membrane coating and the use of MSC-derived vesicle coating to circumvent some of the above-mentioned limitations in the use of OVs. Their principles, advantages, disadvantages, and applications are summarized in Table 2.

**Table 2:** PeptiCRAd, MSC vesicle coating and biomimetic membrane coating.

Platform	Principle	Advantages	Disadvantages	Applications
PeptiCRAd	Synthetic TAAs or patient-specific neoantigens are modified with a positively charged polylysine tail. When mixed with negatively charged adenovirus, the positive tail binds naturally to the negative viral capsid via rapid electrostatic interactions, quickly coating the virus in tumor markers.	Rapid plug-and-play customization Strong dual action (Virus = Adjuvant + Antigen) Overcoming the immunological shield Targeting multiple tumor antigens	Pre-existing immunity due to natural exposure Focus on intratumoral injections Peptide stability and dissociation risk Dependency on hot tumor profile	The primary application is treating advanced solid malignancies that are refractory and resistant to standard checkpoint inhibitors. PeptiCRAd-1 with two cancer-testis antigens: MAGE-A3 and NY-ESO-1 (NCT05492682) is tested in combination with pembrolizumab for advanced, injectable solid tumors like melanoma, triple-negative breast cancer, and NSCLC. Could be used to shorten the manufacturing time for CAR-T. Following tumor antigen identification, the matching peptides are chemically attached to the virus right before administration.
Biomimetic membrane	Cloaks OVs inside natural cell membranes. A synthetic core is wrapped in a natural lipid bilayer keeping the original proteins, lipids, and carbohydrates of the source cells intact. It tricks the body into recognizing the artificial object as a natural cell.	Can be administered intravenously Prolonged blood circulation Enhanced tumor targeting due to natural homing capabilities Minimizes off-target viral infection in healthy organs Membrane can be engineered or chemically modified	Complex manufacturing and scale-up Low yield and reproducibility Risk of contamination by tumor genetic material Coating can disrupt viral infection Regulatory hurdles for clinical trial approval	Treatment of advanced, metastatic, and hard-to-reach solid tumors. Neural/glioblastoma cell membranes are used to coat oncolytic adenoviruses to safely cross the blood–brain barrier and target brain tumors (Jia et al., 2023). In advanced ovarian cancer it increased the tumor-to-off-target virus biodistribution ratio to 36.1 by reducing clearance and fluid interference (Li et al., 2026). Using homologous tumor cell membranes allows the virus to bind to and penetrate pancreatic tumor tissue more efficiently than chemically bound and non-coated viral alternatives (Chen et al., 2024).
MSC-derived vesicles	The mesenchymal stem cell-derived vesicle lipid bilayer cloak hides the viral particles from neutralizing antibodies in the bloodstream, avoiding immune system recognition and supporting tumor cell absorption.	Reduced immunogenicity (neutralizing antibodies) Can be administered intravenously Superior tumor targeting (homing receptors from parent MSCs) High infection efficiency bypassing need for viral receptors Improved safety profile using a cell-free system	Manufacturing complexity involving multi-step processes Low production yield due to isolation methods Heterogeneity in size, cargo, and surface protein density Short *in vivo* half-life (liver, spleen, macrophage clearance) Loading efficiency and inconsistency	Many advanced cancer cells downregulate surface receptors like the coxsackievirus-adenovirus receptor (CAR) to escape infection. Wrapping oncolytic adenoviruses inside MSC vesicles bypasses the need for CAR, enabling much higher internalization rates (Alam et al., 2025). Coating HSV-1 with MSC membranes functions as a cap against pre-existing natural antibodies (Lemos de Matos et al., 2020). Vaccinia virus is a large DNA virus designed to replicate rapidly inside tumors. Encapsulating vaccinia virus in MSC vesicles protects it from fast clearance by the liver and spleen during systemic intravenous delivery (Ramírez et al., 2015).

The main characteristics and advantages of each technology are described.

Another strong candidate for modular incorporation with existing technologies is the creation of a mammalian hyaluronate lyase. In nature, mammalian hyaluronidases are strictly hydrolases, while their prokaryotic counterparts are lyases (Stern et al., 2006; Jiang, J.-Y. et al., 2024; Seo et al., 2026). To benefit from a non-tumor-promoting hyaluronidase compatible with mammalian biology, three main hurdles must be overcome: transcriptional, post-translational, and immunogenicity (Bhatwa et al., 2021; Jiang, R. et al., 2024). Codon optimization, improving mammalian translation, and optimizing protein structure for complex mammalian environments are greatly facilitated by modern AI-driven tools (Chen et al., 2025; Fu et al., 2026).

## 6 Challenges and future directions

Amid OVT’s hurdles (off-target infection risks, limited penetration through the ECM of cold tumors, and limited efficacy due to immunosuppression), phage-derived components offer an attractive solution, but not without hurdles. Phage hybrids can face vector instability, and anti-phage antibodies combined with a lack of replication can shorten half-life. Regulatory concerns could delay trials, and preclinical ones do not fully account for human heterogeneity and are predominantly in murine models. Still, the potential of bridging phage and virus characteristics could significantly help bypass systemic hurdles and reshape the tumor microenvironment. The selection of the best phage-OV strategy to combat tumors depends on the tumor type, route of administration, and treatment stage. In the case of immunologically “cold” tumors, it would be relevant to prioritize the integration of highly immunogenic phage capsid proteins (e.g., T4) into the OV genome as they serve as strong pathogen-associated molecular patterns (PAMPs) that could engage TLRs to convert the tumor to a “hot” profile. By recruiting dendritic cells and promoting antigen presentation, it can act as a natural adjuvant to reverse immunosuppression as proposed in the case of colorectal cancer (Pérez-Domínguez et al., 2025). Another important aspect discussed in this review is the extracellular matrix. Tumors with a dense ECM (pancreatic, breast, and glioblastoma) restrict standard OV diffusion and penetration. Engineering OVs to express enzymes such as hyaluronidase to degrade the tumor stroma (Lee et al., 2010), facilitating deep viral penetration and improving the diffusion of co-administered chemotherapies, could be one solution. Alternatively, screening for peptides targeting tumor-associated fibroblasts could also be a strategy. The route of administration is highly dependent on the stability of the therapeutic agent and its ability to reach as deep as possible into the tumor. The neutralization by circulating antiviral neutralizing antibodies and liver sequestration by Kupffer cell uptake are the main challenges for intravenous administration. With the advent of phage versatility, it will be possible to adapt the engineering strategy to bypass these issues. This includes swapping hypervariable loop regions of the OV capsid with structurally inert phage capsid fragments to act as an immunological cloak. Intratumoral administration could benefit from the integration of specific phage tail fiber proteins to ensure that, once the OV is injected, it anchors tightly to tumor-specific receptors, preventing systemic leakage and protecting adjacent healthy tissue (Zhu et al., 2024). Finally, the treatment stage significantly influences which phage-OV strategy to adopt. For early stages, and prior to surgery and radiation, the priority is to maximize tumor cell killing, breakdown of the ECM, release of tumor-associated antigens and other danger signals for innate immunity that altogether will support immunological memory. This strategy should support better penetration of subsequent therapeutic rounds (Muthukutty and Yoo, 2023). The focus will differ for later stages of treatment such as during maintenance, tracking of metastasis and minimal residual disease (Wu, P. et al., 2010, Zhu et al., 2024). As these rounds of therapeutics may last longer, the priority is to extend the bioavailability of the phage-OV construct to maximize the recirculation to reach metastasis at the deeper sites of the body. The potency of OV can be increased by expressing a library of patient-specific neoantigens, which then act as an “*in situ* vaccine,” utilizing the adjuvant nature of the phage proteins to drive robust, long-term CD8+ T-cell memory. This should support efficient clearance and control of the few residual cancer cells.

There is a rising number of studies and preclinical trials testing phage/OV hybrids in human cells with optimistic results (Supplementary Table S1). 2025 trials of AAVP hybrids in melanoma/ovarian models herald this, urging interdisciplinary efforts for scalable, personalized therapies that unleash OVT’s full arsenal against immunosuppression. Future research should focus on improving the vector stability of phages, allowing for enhanced overall function and mitigating one of the main limitations of phage therapy and the incorporation of phage mechanisms in OVT. Studies should also explore the possibility of introducing lysis and replication capabilities to OV/phage hybrids, allowing for the best strengths of both OVT and phages to be fully represented in one treatment. Finally, future studies should focus on expanding the diversity of cancer types and phage/OV hybrids included in pre-clinical studies. A more comprehensive exploration of phage-derived mechanisms in diverse cancer types is crucial. This would allow us to overcome current barriers and advance this novel therapeutic approach to clinical trials and eventually transform the immunotherapeutic landscape with this promising method. In conclusion, harnessing bacteriophage-derived components such as phage display and superior delivery capabilities has the potential to allow OVT to transcend TME immunosuppression. By combining greater safety, improved delivery for TME modulation, and greater adaptive anti-tumor immune responses, phages can counter cold tumor barriers and mitigate challenges faced by OVs. While challenges such as vector instability and regulatory hurdles remain, the convergence of phage and oncolytic virotherapy represents a critical frontier in oncology. This is an active field of interdisciplinary study with the potential to transform immunotherapy, making it safer, more effective, less toxic, and more accessible.
